# Effects of ketone bodies on energy expenditure, substrate utilization, and energy intake in humans

**DOI:** 10.1016/j.jlr.2023.100442

**Published:** 2023-09-11

**Authors:** Rodrigo Fernández-Verdejo, Jacob T. Mey, Eric Ravussin

**Affiliations:** 1 Pennington Biomedical Research Center, Louisiana State University, Baton Rouge, LA, USA; 2 Laboratorio de Fisiología del Ejercicio y Metabolismo (LABFEM), Escuela de Kinesiología, Facultad de Medicina, Universidad Finis Terrae, Santiago, Chile

**Keywords:** obesity, nutrition, dietary fat, lipids/oxidation, insulin, ketosis, beta-hydroxybutyrate, appetite, energy balance

## Abstract

The potential of ketogenic approaches to regulate energy balance has recently gained attention since ketones may influence both energy expenditure and energy intake. In this narrative review, we summarized the most relevant evidence about the role of ketosis on energy expenditure, substrate utilization, and energy intake in humans. We considered different strategies to induce ketosis, such as fasting, dietary manipulation, and exogenous ketone sources. In general, ketosis does not have a major influence on energy expenditure but promotes a shift in substrate utilization towards ketone body oxidation. The strategies to induce ketosis by reduction of dietary carbohydrate availability (e.g., ketogenic diets) do not independently influence energy intake, being thus equally effective for weight loss as diets with higher carbohydrate content. In contrast, the intake of medium-chain triglycerides and ketone esters induces ketosis and appears to increase energy expenditure and reduce energy intake in the context of high carbohydrate availability. These latter strategies lead to slightly enhanced weight loss. Unfortunately, distinguishing the effects of the various ketogenic strategies per se from the effects of other physiological responses is not possible with the available human data. Highly controlled, inpatient studies using targeted strategies to isolate the independent effects of ketones are required to adequately address this knowledge gap.

Ketosis describes the metabolic state in which circulating ketone bodies elevate beyond normal levels. Over the past decade, strategies aiming to induce ketosis have gained interest in research and public sectors for their potential role in body weight management. Different nutritional interventions are used as such strategies. Ketogenic diets (KDs) represent a classic example, which is based on decreasing carbohydrate availability to boost hepatic ketogenesis. Following the directive of “do not eat carbohydrates”, KDs are purported to increase “fat burning” (oxidation) and reduce hunger cravings because of reduced insulin levels. Thus, diet-induced ketosis may be highly appealing to individuals struggling with obesity. In a world with rampant obesity and a multibillion-dollar weight loss industry, understanding the role of ketosis in weight management is both scientifically essential and economically lucrative. Whether the purported effects of ketosis persist through the rigors of the scientific approach and play a role in weight management is the focus of this narrative review.

Weight management in the context of obesity involves weight loss followed by sustaining that weight loss (weight maintenance). It is well established that weight loss is caused by sustained negative energy balance, whereby energy expenditure is greater than energy intake. If the negative energy balance concurs with enhanced fat oxidation, this should favor fat mass loss and potentially confer enhanced health benefits. Toward this, an intense scientific debate has emerged over the past few years demonizing carbohydrates and insulin, theorizing that they play causal roles in the pathological accumulation of body fat (1, 2, 3, 4, 5, 6, 7). According to the “carbohydrate-insulin model” of obesity, an increased proportion of rapidly digestible carbohydrates (i.e., a high glycemic load) in the diet elevates insulin secretion, thus suppressing the release of fatty acids into circulation and directing circulating fat toward storage (6). Metabolically active tissues would perceive the decreased availability of circulating fatty acids (and glucose) as a state of cellular internal starvation. This state would then decrease energy expenditure and increase food intake as an adaptive mechanism (4, 8, 9, 10). Based on this model, decreasing the dietary carbohydrate-to-fat ratio without altering protein should reduce insulin secretion, increase fat mobilization, and enhance the oxidation of circulating fatty acids. The resulting metabolic and endocrine milieu would relieve the state of cellular internal starvation, thus decreasing hunger, increasing energy expenditure, and promoting weight loss. However, most randomized clinical trials in humans suggest that manipulating the carbohydrate-to-fat ratio in the diet has no independent impact on body weight. Both KDs (high fat, low carbohydrate) and non-KDs (e.g., high carbohydrate, low fat) induce equivalent weight and fat loss (11, 12), although some research programs suggest ketogenic approaches confer unique weight loss benefits (13). A critical differentiating factor between KDs and non-KDs is the increased production of ketone bodies and persistently elevated plasma ketone concentrations (i.e., ketosis). Understanding the biological effects of ketones on energy expenditure and energy intake may thus help elucidate the potential effects of ketogenic approaches on energy balance and weight control. To that end, some confounding factors must be considered.

A first factor when interpreting the effects of ketosis on energy balance is the operational definition of “ketosis” itself. Circulating plasma ketones in healthy conditions are typically ∼80% beta-hydroxybutyrate (ßOHB), ∼20% acetoacetate, and <1% acetone, with the ßOHB-to-acetoacetate ratio approaching 1:1 during substantial ketosis (14, 15). Even so, ketosis is often assessed by the circulating concentrations of ßOHB. Currently, there is no real consensus definition of ketosis and therefore no threshold for consistently characterizing ketosis between reports. The circulating concentrations of ßOHB considered to be indicative of ketosis vary widely. A circulating concentration lower than 0.5 mM is generally considered normal (16), with values after an overnight fast often ranging between 0.02 and 0.3 mM. Ketosis goals of nutrition therapies to treat refractory epilepsy or status epilepticus may reach circulating concentrations of ßOHB between 2.0 and 5.0 mM (17). Nevertheless, for purposes such as weight loss, ketosis goals may include wider ranges. Weight loss trials have used an arbitrary state of "nutritional ketosis" defined as circulating ketone concentrations (usually circulating ßOHB) between 0.5 and 3.0 mM (18, 19, 20).

A second confounding factor when interpreting the impact of ketosis on energy balance is the method used to induce ketosis. Nutritional strategies that reduce carbohydrate availability concomitantly increase fat oxidation, thus stimulating hepatic ketogenesis. One commonly accepted strategy is to consume a KD, which provides <50 g/day or 5%–10% of energy intake as carbohydrates (21). Importantly, KDs allow us to study the effects of ketosis while often maintaining energy balance. Another strategy is the use of a very-low-calorie ketogenic diet (VLCKD), characterized by very low energy and carbohydrate intake accompanied by adequate protein, essential fatty acids, and micronutrient intake. VLCKDs were initially proposed as a protein-sparing therapy for conditions associated with caloric deprivation (22). For example, a 2–3 weeks weight reduction program supplying 1.0 g protein/kg body weight per day was adequate to maintain nitrogen balance while achieving circulating ketone concentrations between ∼1.0 and 4.0 mM (23). Subsequent outpatient studies have demonstrated that VLCKDs significantly increase circulating ketone concentrations (24, 25, 26, 27). Current guidelines to implement VLCKDs include providing 500–800 kcal/day, <50 g carbohydrate/day, and 1.0–1.5 g protein/kg body weight per day (28). Finally, an extreme strategy to induce profound ketosis is prolonged fasting. Compared to a 10–12 h fast, a 20 h (29), 48 h (30), 60 h (31), and 72 h (32) fast progressively increase the circulating concentrations of ßOHB, reaching up to ∼3.0 mM. Nevertheless, even if prolonged fasting strongly triggers ketosis, this paradigm may be irrelevant to assess the impact of ketones on energy expenditure (and of course energy intake) as there is no comparison with fed individuals. A comparison can only be assessed between various levels of ketosis. Importantly, the disadvantage of KDs, VLCKDs, and prolonged fasting is that they all produce multiple metabolic responses besides increasing circulating ketones. Whether ketones independently mediate the effects on energy expenditure and energy intake cannot be truly ascertained from these dietary interventions alone.

Additional ketogenic strategies can increase circulating ketones in the context of substantial carbohydrate availability. Such strategies thus allow for the assessment of the physiologic effects of ketones with fewer dietary confounding factors than traditional KDs or VLCKDs. The consumption of medium-chain triglycerides (MCTs) is one of those strategies. MCTs are composed of fatty acid chains of 6–12 carbons in length. Such fatty acids bypass lymphatic transport, thus being directly transported to the liver through the portal vein. In the liver, MCTs are readily oxidized, as they can freely enter the mitochondrial matrix, independent of the carnitine shuttle (33). This rapidly produces excess acetyl-CoA and induces hepatic ketogenesis, after which ketones (which cannot be oxidized in the liver) are exported into the circulation (for a review see (34)). As a result, circulating concentrations of ßOHB transiently increase after MCT ingestion, but not after long-chain triglyceride (LCT) ingestion (35, 36, 37). A second strategy to increase ketones in the context of substantial carbohydrate availability is by the consumption of exogenous ketone supplements, most commonly ketone esters. This strategy rapidly increases the circulating concentrations of ßOHB (38). Finally, a third strategy to induce ketosis independent of dietary changes is through the intravenous infusion of ketones. Although not a viable real-world approach, the ability to manipulate circulating ketones directly and independent of the diet is critically valuable in a research and mechanistic context.

Overall, each strategy for inducing ketosis provides valuable information to understand the effect of ketone bodies on the components of energy balance. Nevertheless, the advantages and disadvantages of each strategy should be considered when interpreting the findings. This review discusses the evidence of the effects of ketone bodies on energy expenditure, substrate utilization, and energy intake in humans.

## Impact of Ketosis on Energy Expenditure and Substrate Utilization

### Ketogenic diets

The effects of KDs and other low-carbohydrate diets on weight management have been historically refuted (5, 6, 7). Although these diets are now supported as effective clinical approaches to weight management, whether they are more effective than nonketogenic approaches remains controversial. Part of the debate involves their effects on energy expenditure. Despite dedicated research efforts, differences in study designs (inpatient vs. outpatient), assessment methods (whole-body room calorimetry vs. doubly labeled water), and calculations (whether or not to correct for the respiratory exchange ratio [RER]) have triggered contentious scientific disputes (39, 40, 41, 42, 43). In this section, we discuss some of the most relevant studies investigating the impact of ketosis on energy expenditure and substrate utilization.

We conducted an inpatient study to determine the effect of transitioning from a standard high-carbohydrate diet to a KD on energy metabolism using both whole-body room calorimetry and doubly labeled water (39, 41). Men were fed a 4-weeks baseline diet (50% and 35% of energy from carbohydrates and fat, respectively) followed by an isocaloric 4-weeks KD (5% and 80% of energy from carbohydrates and fat, respectively). The KD increased the circulating acetoacetate and ßOHB to values consistent with nutritional ketosis (0.781 mM and 0.758 mM, respectively) (18, 19, 20). During the first week of the KD, the 24-h RER dropped from 0.88 to 0.78 and then remained constant indicating increases in fat oxidation (RER = 0.71), ketogenesis (RER = 0), and endogenous ketone oxidation (RER value as the fatty acid precursor) (44, 45). Total energy expenditure (TEE) measured by room calorimetry transiently increased by ∼100 kcal/day during the first two weeks of the KD but then returned to the values measured in the baseline diet. Exploratory doubly labeled water analyses initially suggested that TEE had remained elevated during the last two weeks of the KD (39, 40, 42). Nevertheless, the response was not evident when RER calculations accounted for the effect of the diet and energy balance (participants were unintentionally in negative energy balance) (43, 46). In response to the KD, changes in sleeping metabolic rate mostly accounted for the transient increase in TEE. Awake and fed thermogenesis was not different between diets, while physical activity energy expenditure was lower with the KD. Similar results were observed in a subsequent inpatient crossover study conducted by Hall *et al.* (47). Therein, men and women were randomly fed ad libitum a low-fat diet (75% and 10% of energy from carbohydrates and fat, respectively) and a KD (10% and 75% of energy from carbohydrates and fat, respectively) for 2 weeks. Again, energy expenditure was measured using whole-body room calorimetry. The KD (vs. low-fat diet) was associated with higher circulating ketones (3.01 vs. 0.21 mM), and lower RER (0.75 vs. 0.88). TEE was 153 kcal/day higher in the KD than in the low-fat diet, mostly due to differences in resting energy expenditure (REE). Consequently, basal metabolic processes seem to underlie the transient increases in the TEE induced by KDs. An increase in gluconeogenesis while the brain shifts from glucose to ketone utilization may partially explain the increased energy expenditure (48).

The effect of KDs on energy metabolism has also been tested during weight-loss maintenance. Ebbeling *et al.* (49) conducted a 3-way crossover outpatient study wherein participants were first exposed to a 12-weeks weight loss diet. Then, participants were randomized to 4-weeks isoenergetic weight-loss maintenance diets with low fat (60%, 20%, and 20% of energy from carbohydrates, fat, and protein, respectively), low glycemic index (40%, 40%, and 20% of energy from carbohydrates, fat, and protein, respectively), or very-low-carbohydrate (10%, 60%, and 30% of energy from carbohydrates, fat, and protein, respectively). Although circulating ketones were not reported, the very-low-carbohydrate diet could be considered as a KD based on the proportion of energy from carbohydrates. TEE was measured by doubly labeled water during the last two weeks of the diets, and REE by indirect calorimetry in the last week. Weight loss decreased TEE and REE, but during weight maintenance, TEE remained ∼300 kcal/day and REE ∼67 kcal/day higher on the KD compared to the low-fat diet. A sustained increase in gluconeogenesis may explain the higher REE and part of the higher TEE with the KD (48). Physical activity energy expenditure seems implausible because objectively measured physical activity was not different between diets. The KD may have induced an elevated thermic effect of food (TEF) related to its higher protein content (30% vs. 20% in the other diets) (50). Nevertheless, the most likely explanation for the higher TEE in the KD relates to diet-specific energy imbalance effects on RER unaccounted for in calculations (46). Of note, although energy intake (2626 kcal/day) was ∼160–450 kcal/day lower than TEE during the weight loss maintenance, body weight did not seem to decrease. This suggests that either TEE measurements were inaccurate, participants were nonadherent to the diets, or both. As for RER, the KD showed the lowest values, consistent with higher fat oxidation, ketogenesis, and endogenous ketone oxidation (44, 45).

The evidence demonstrates that short-term KDs stimulate fat oxidation and produce a transient, small increase in REE. In contrast, the large effects of KDs (and other less extreme low-carbohydrate diets) on TEE (39, 42, 49, 51) seem to derive from unadjusted calculations of doubly labeled water data (43, 46, 52). The effects of KDs on energy metabolism are, however, proposed to be more clearly manifested after several weeks of intervention (53). Unfortunately, long-term studies have not been conducted to confirm or refute this hypothesis. Besides the tedious, costly, and imprecise approach of the intake balance method using doubly labeled water and body composition (54), there are no objective methods to precisely measure energy intake in free-living individuals (55, 56). Although some outpatient studies provide all the foods to participants (49, 51), the most commonly utilized approach is conducted by providing diet prescriptions along with lifestyle and behavioral guidance to participants to modify their habitual diet. In these outpatient settings, adherence is difficult to assess and often modest (57). Thus, the majority of outpatient studies are deemed to be testing the effectiveness of diet prescriptions and behavioral interventions instead of the physiologic effect of altering dietary intake per se (58). Long-term, adequately powered, inpatient studies are required to directly test the effects of KDs on energy metabolism. As described by Hall (59), such studies are expensive and labor-intensive, but not impossible. Alternatively, the long-term effects of KDs can be mathematically modeled by assuming full adherence to the diet prescription. Using this approach, the decrease in TEE induced by a 6-months 30%-calorie restriction was shown to be similar between a KD (5%–10% of energy intake from carbohydrates) and a mixed diet (55% of energy intake from carbohydrates) (58). Recent reviews identified several studies analyzing the effects of KDs on energy expenditure (12, 19, 60). Yet considering the methodological aspects previously discussed, the conclusions of individual studies must be interpreted with caution.

### Very-low-calorie KDs

Evidence for the effects of VLCKDs on energy metabolism is limited. Gomez-Arbelaez *et al.* (27) investigated the effects of a 90-days VLCKD on REE in humans. REE was measured by indirect calorimetry using only O_2_ consumption. Compared to the baseline, the VLCKD reduced REE by ∼20%. Similar to previous caloric restrictions with or without exercise, approximately 25% of weight lost was fat-free mass (61). Also, the VLCKD increased the circulating concentrations of ßOHB to ∼0.7–1.9 mM, consistent with nutritional ketosis (18, 19, 20). REE showed a nonsignificant decrease which was not different from the expected values considering the loss of fat-free mass. The VLCKD thus prevented the larger-than-expected decrease in REE consistently observed in weight loss interventions, even when fat-free mass is preserved (62). Since ßOHB concentration was not a significant determinant of REE, the effects of the VLCKD seemed independent of ketosis. These findings must, however, be interpreted with caution due to methodological confounders including an outpatient design (58); the assumption of an RER of 0.85, which is unlikely for a ketogenic, negative energy balance condition (44, 46, 58, 63, 64); a calculation of predicted REE that assumes that REE and fat-free mass are directly proportional, which is not typically observed (62, 65, 66); the absence of a non-KD control group; and the inclusion of lifestyle counseling with instructions to exercise regularly. Consequently, the effects of VLCKDs on energy metabolism in humans remain to be determined.

### Prolonged fasting

Small increases in REE (∼5-6%) have been reported after 20 h and 72 h of fasting (29, 32), with a similar nonsignificant trend after 48 h (30). Prolonged fasting has also been shown to decrease RER, indicating enhanced fat utilization (29, 30, 32). Highly controlled inpatient studies conducted using whole-body room calorimetry confirm the effects of prolonged fasting on RER. Compared to conditions of energy balance, a 24-h fast was associated with lower sleeping and 24-h RER (67), while a 48-h fast was associated with progressively lower 24-h RER values over time (68). In both studies, TEE was ∼8–9% lower during prolonged fasting conditions likely due to the absence of TEF (67, 68), while sleeping energy expenditure was unaffected during a 24-h fast (67) but decreased by ∼4% (*P* = 0.05) during a 48-h fast (68). These data show small effects of prolonged fasting on sleeping EE (or REE). Notably, these effects are within the range of the circadian variability for REE (69). In contrast, prolonged fasting consistently decreases RER beyond what can be explained by circadian variability (69). This response indicates increased fat oxidation, ketogenesis, and endogenous ketone oxidation (44, 45) as ketones become a major substrate for the brain during periods of low carbohydrate availability (70).

**Fig. 1:**
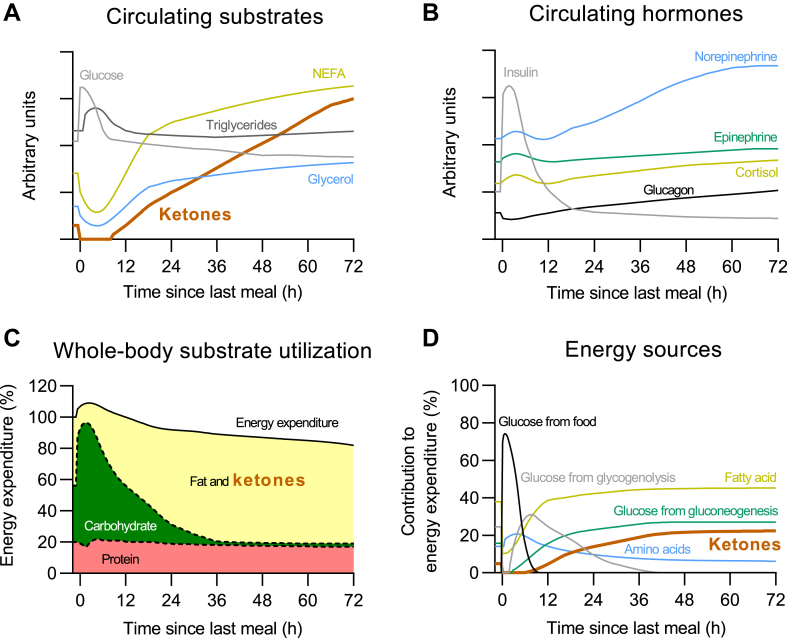
Effects of prolonged fasting in (A) circulating substrates, (B) circulating hormones, (C) whole-body substrate utilization, and (D) energy sources in humans. NEFA, non-esterified fatty acids. Modified from Dote-Montero *et al. Nutrients* 14, 2022.

Recent nutritional strategies for improving metabolic health include intermittent periods of prolonged fasting (71, 72). Circulating ketones increase during prolonged fasting but rapidly decrease upon refeeding (73). The transient increase in ketones may mediate some of the hypothesized health benefits of these nutritional interventions, but this is yet to be demonstrated. Indeed, like KDs and VLCKDs, prolonged fasting not only increases circulating ketones but also influences the concentrations of many other energy substrates and hormones (27, 29, 30, 31, 32, 39, 49, 74) (Fig. 1). Dote-Montero *et al.* (72) recently summarized most studies about the effects of intermittent fasting strategies on energy metabolism in humans. They concluded that intermittent fasting enhances fat oxidation but induces similar effects on energy expenditure compared to caloric restriction. Notably, the physiological effects triggered by intermittent fasting strategies will depend on the timing of the measurements during the fasting days versus the fed days.

### Administration of MCTs and intravenous infusion of ketones

Some well-controlled studies have provided insight into the effects of MCT intake on energy metabolism in humans. Seaton *et al.* (35) conducted an inpatient study to compare the effect of MCTs and LCTs on energy metabolism. Following a standard dinner and an overnight fast, participants were fed either a meal containing 48 g of MCTs or 45 g of LCTs. Energy metabolism was measured by indirect calorimetry over 6 h after the meal. Circulating ßOHB increased to ∼0.6 mM with the MCTs, but it did not change with the LCTs. The TEF was 3-fold larger after the MCTs, whereas the RER decreased similarly from ∼0.85 to 0.78 with both meals. These observations are supported by a double-blind, crossover, randomized study using whole-body room calorimetry. Therein, Dulloo *et al.* (75) fed participants with isocaloric diets supplemented by different MCT/LCT ratios for 24 h (0/30, 5/25, 15/15, 30/0 g). Although circulating ketones were not measured, progressively increasing the amount of MCTs led to higher TEE. Thus, TEE was ∼113 kcal/day higher with a 30-g intake of MCTs compared to no MCTs (30/0 vs. 0/30 g conditions, respectively). The ratio of MCT/LCT neither influenced RER nor urinary nitrogen. Together, these data suggest that acute intake of MCTs stimulates ketogenesis and energy expenditure (TEF specifically) without affecting relative substrate oxidation.

Determining the sustained effects of MCTs on energy metabolism is, however, challenging. It requires long-term, adequately powered, inpatient studies. St-Onge *et al.* (76) conducted one of the most controlled studies to date. In a randomized crossover design, women were fed diets rich (75% of total fat) in MCTs or LCTs for 27 days. The women resided in a research unit, and all food was consumed under supervision, but participants could leave during the day for work or classes. REE along with the TEF and fat oxidation (over 6 h after a breakfast with MCTs or LCTs) were measured by indirect calorimetry on the 2nd and 27th day. REE was not different between diets. On both the 2nd and 27th day, the diet rich in MCTs produced a slightly higher TEF (by 7.2 kcal) and absolute fat oxidation (by 1.6 g). Both diets decreased body weight to a similar extent without influencing body composition. Therefore, the increased TEF induced by MCTs did not influence body weight. This could be due to the magnitude of TEF being too small to observe an effect on body weight within the 4-weeks study. Alternatively, other components of energy expenditure (or intake) may have undergone a compensatory regulation to account for the TEF difference. Note that circulating ketone concentrations were not reported.

Finally, the most direct approach to studying the metabolic effects of ketones in humans is through intravenous infusions. Chioléro *et al.* (77) analyzed the effect of a continuous intravenous infusion of sodium ßOHB for 3 h in humans. Note that while ßOHB has an RER of 0.89, sodium ßOHB has an RER of 0.66 (78). During the last hour of infusion, circulating ßOHB was 0.94 mM, thus reaching the range proposed for nutritional ketosis (18, 19, 20). The REE increased by 5% while RER dropped due to increased oxidation of ßOHB concomitant with decreased oxidation of endogenous lipids and carbohydrates (−77% and −25%, respectively). This suggested that exogenous ßOHB served as an energy substrate that displaced the oxidation of endogenous substrates. Nevertheless, in the absence of a control condition, the effects of the infusion cannot be distinguished from those of time. In contrast, Nair *et al.* (79) compared the effects of 8-h intravenous infusions of ßOHB versus saline on gas exchange (indirect calorimetry) and leucine oxidation (labeled isotopes). The ßOHB infusion increased circulating ßOHB up to ∼2.0 mM, whereas the saline infusion had no effect. The infusion of ßOHB was associated with lower leucine oxidation and higher protein synthesis in skeletal muscle. O_2_ consumption and CO_2_ production were, however, not different between ßOHB and saline infusions. Together, these studies of intravenous infusions of ketones conducted in the 1980s and 1990s did not show a clear increase in energy expenditure but showed a redistribution of endogenous substrate oxidation. These data are in agreement with a recent study that tested the effects of growth hormone infusions along with either ßOHB or saline in men (80). Therein, circulating ßOHB increased to ∼3.3 mM after 180 min of ßOHB infusion but had a minimal increase after saline infusion. ßOHB infusion neither affected REE nor RER. During an euglycemic–hyperinsulinemic clamp, however, energy expenditure increased more while RER trended (*P* = 0.06) to increase less during ßOHB infusion compared to saline. This suggests an interaction between ßOHB, insulin, and growth hormone that may influence energy metabolism.

### An integrative view of the effects of ketone bodies on energy expenditure and substrate utilization

Table 1 summarizes the evidence for the effects of KDs, VLCKDs, prolonged fasting, MCTs, ketone esters, and ketone infusions on energy expenditure and substrate utilization in humans. Mechanistically, KDs, VLCKDs, and prolonged fasting are characterized by a reduction in carbohydrate availability that promotes an increased dependence on lipids and proteins as energy sources. The circulating concentrations of nonesterified fatty acids, glycerol, and glucagon are increased, whereas the circulating concentrations of insulin are reduced (23, 29, 30, 31, 32, 39). Together, these metabolic and hormonal changes stimulate liver gluconeogenesis to maintain glucose supply to the brain (for a review see (81)). The metabolic pathways involved in gluconeogenesis are energetically costly and underlie part of the increase in REE induced by reduced carbohydrate availability (48). Concomitant with gluconeogenesis, increased fat oxidation in the liver stimulates ketogenesis. Circulating ketones then serve as an alternative energy substrate for the brain (70), thus reducing the need for gluconeogenesis and partially explaining the transient nature of the increase in REE induced by KDs (39, 43, 46). Intravenous infusions of ßOHB have been shown to decrease endogenous glucose production in overnight fasted individuals (82, 83). Consequently, ketone bodies are part of the physiological response in REE induced by reduced dietary carbohydrate availability. Ketone bodies are also implicated in the changes in whole-body substrate utilization. The increased whole-body fat oxidation (RER = 0.71), liver ketogenesis (RER = 0), and ketone oxidation in the brain and skeletal muscle all underlie the drop in whole-body RER (44, 45, 70, 83). Data from intravenous infusions also suggest that of ßOHB decreases amino acid oxidation, which could help preserve muscle mass (79). This last observation is contrary to the more negative nitrogen balance observed in KDs compared to non-KDs (39, 47). Nevertheless, this controversy may be explained by other metabolic changes induced by the KDs instead of the increase in circulating ketones.

**Table 1:** Strategies to increase circulating ketones and their effect on energy expenditure, substrate utilization, and energy intake in humans

Strategy	Context			*Component Related to the Regulation of Energy Balance*						
	CHO Availability	Energy Balance	Ketones Source	REE	TEF	PAEE	TEE	RER	Appetite	Energy Intake
Ketogenic diet	↓	Null or negative	Endo	↑ (transiently)	?	↓	↑ (transiently)	↓	⟷	⟷ or ↑
Very-low-calorie ketogenic diet	↓	Negative	Endo	↑ (probably)	?	?	↑ (probably)	↓ (probably)	⟷ (probably)	⟷ or ↑ (probably)
Prolonged fasting	↓↓	Very negative	Endo	↑	↓↓	?	↓	↓↓	NA	NA
Intake of medium-chain triglycerides	⟷	Null	Endo	⟷	↑	?	↑	⟷^a^	⟷	↓^c^
Intake of ketone esters	⟷	Null	Exo	?	?	?	?	?	↓ (probably)	?
Intravenous infusion of ßOHB	⟷	Null	Exo	⟷ (probably)	NA	NA	⟷ (probably)	⟷ or ↓^b^	?	?

↑, increases; ↓, decreases; ⟷, no change; ßOHB, beta-hydroxybutyrate; REE, resting energy expenditure; TEF, thermic effect of food; PAEE, physical activity energy expenditure; TEE, total energy expenditure; RER, respiratory exchange ratio; Endo, endogenous; Exo, exogenous; NA, not applicable.

a When comparing medium-chain to long-chain triglycerides.

b Note that although the RER of ßOHB is 0.89, the RER of the infusate sodium ßOHB is 0.66.

c When comparing medium-chain to long-chain triglycerides in contexts of high carbohydrate availability.

These physiological responses are consistent with the data from intravenous infusions of ketone precursors or ketones. Since carbohydrate availability is not affected by such infusions, gluconeogenesis is not stimulated, and REE does not seem to increase. The ingestion of MCTs produces an increased TEF for the digestion, absorption, and oxidation of triglycerides. MCT ingestion causes a drop in RER like that of LCT ingestion, suggesting that the response is mostly driven by increased fat oxidation.

Finally, ketone bodies influence substrate utilization by modifying the circulating concentrations of many energy substrates. Intravenous infusions of ßOHB decrease the circulating concentrations of glucose (77, 79, 82, 83, 84), nonesterified free fatty acids (77, 79, 82, 83, 84), glycerol (83), and amino acids (79, 82), while decreasing the appearance rate of glucose (82, 83) and glycerol (83). The effect of ßOHB on glucose is probably mediated by the inhibition of gluconeogenesis, while the effect on amino acids is related to the inhibition of protein degradation and activation of protein synthesis in skeletal muscle. The molecular mechanisms are, however, unknown. The effect of ßOHB on nonesterified fatty acids and glycerol likely results from the inhibition of adipose tissue lipolysis through a receptor-mediated mechanism. In vitro studies showed that ßOHB is a ligand for the G-protein coupled receptor 109A (85) with an EC_50_ of 0.77 mM, which is within the range of ßOHB concentrations observed in nutritional ketosis (18, 19, 20). In mouse adipocytes, ßOHB attenuates the efflux of nonesterified fatty acids induced by isoproterenol (85). Therefore, in conditions of nutritional ketosis, ßOHB may attenuate the release of nonesterified fatty acids and glycerol from adipose tissue and thus decrease liver gluconeogenesis and ketogenesis. The fact that infusion of ßOHB inhibits the endogenous appearance of ßOHB supports the presence of a negative feedback loop (83).

## Impact of Ketosis on Energy Intake

### Ketogenic diets

One of the core tenants to reducing energy intake in humans is to reduce appetite (86). KDs have been used to study the effects of ketosis on appetite and energy intake. Importantly, using KDs as a model to understand the effect of ketosis on appetite and energy intake is difficult, as this method is often conducted in the context of weight loss. This typically involves intentional reductions in energy intake, profound changes in dietary patterns, and is often combined with intensive behavioral therapy. Also, KDs may reduce hedonic eating from emotional or other external stimuli (87). These factors represent substantial confounding towards understanding the role of ketosis in energy intake as they may independently impact appetite and energy intake along with body weight. With this in consideration, herein we discuss some of the most relevant evidence for the effect of KDs on energy intake.

One of the most well-controlled feeding trials investigating the effect of ketosis on energy intake was the previously mentioned inpatient feeding study conducted by Hall *et al.* (47). The authors compared the ad libitum energy intake during a KD and a non-KD in 20 men and women with a weight status ranging from normal weight to obesity. Participants were randomized to consume a KD (10% and 75% of energy from carbohydrates and fat, respectively) or a non-KD (75% and 10% of energy from carbohydrates and fat, respectively) for 2 weeks, followed immediately by the alternate diet for another 2 weeks. Importantly, study participants were informed to eat “normally” without the intention of weight loss and were presented with ample food availability. Participants spontaneously lost modest (∼1–2 kg) but similar amounts of weight on both diets. Together, this minimizes confounding from intentional reductions in energy intake and removes the confounding from concomitant weight loss strategies such as intensive behavioral therapy. The KD increased circulating ßOHB to ∼2.0 mM and resulted in increased energy intake (∼550–700 kcal/day) without a difference in self-reported appetite between diets. This led to the conclusion that unlike hypothesized, a KD did not reduce acute energy intake or appetite. Over the 2-weeks KD, energy intake was ∼300 kcal/day lower in the second week compared to the first week, consistent with elevated and stabilized plasma ßOHB. This is noteworthy because ßOHB concentrations during the first week on the KD steadily increased from baseline (∼0.5 mM) and reached ∼2 mM only at the start of the second week. Comparing energy intake during the second week between the KD and non-KD, energy intake still remained significantly lower on the non-KD, but it is unclear whether a sustained elevation in ßOHB would continue to reduce energy intake on the KD over a longer period of time. As identified by the authors, one of the limitations of the study was the difference in energy density between diets (∼2 kcal/g for KD vs. ∼1 kcal/g for non-KD), as it is well established that food energy density is an independent factor determining total energy intake (88, 89). Nevertheless, the confounder of caloric density is difficult to experimentally address because of the inherent difference in caloric density between fat (9 kcal/g) and carbohydrate (4 kcal/g). Hengist *et al.* (90) conducted another short-term study (24 h) that matched three different diets for energy density, one of which elicited a modest ketogenic response (∼0.5 mM). The study used an open-label, multiple-crossover design to assess ad libitum energy intake during a monitored lunch meal and a free-living dinner meal. No differences in energy intake were observed over 24 h, suggesting that modest KD-induced ketosis does not influence energy intake when the energy density is controlled. In conclusion, KD-induced ketosis does not likely independently influence energy intake when comparing KD and non-KD approaches during weight stability.

Since the real-world application of KDs and non-KDs is most often to induce long-term weight loss, investigating the effect of KD-induced ketosis on energy intake and appetite during weight loss interventions is of particular interest. The effect of diet on appetite is important in the setting of long-term weight loss studies because progressive weight loss leads to increased hunger and subsequently increased energy intake (both drivers of weight regain/weight loss recidivism). Johnstone *et al.* (91) conducted one of the most well-controlled feeding trials investigating the effect of ketosis on energy intake during weight loss. The study was a resident, ad libitum feeding trial wherein men with obesity lived on a research unit and consumed only study foods but were able to leave the unit for work. Participants were randomized to consume either a KD (4% and 66% of energy from carbohydrates and fat, respectively) or a non-KD (35% and 35% of energy from carbohydrates and fat, respectively) for 4 weeks, followed by the alternate diet for another 4 weeks. A 3-days maintenance diet was provided before the study, between the diets, and at the end of the study. The entire study lasted ∼9.5 weeks. Participants lost weight on both diets but significantly more on the KD diet compared to the non-KD diet (6 kg or 5.8% vs. 4 kg or 4.0%, respectively). This corresponded with reduced energy intake (167 kcal/day) and lower feelings of hunger on the KD concomitant with elevations in ßOHB from ∼0.2 mM at baseline to 1.5 mM after treatment (compared to ∼0.3 mM at baseline and after treatment on the non-KD). The authors concluded that the KD reduced energy intake without increasing hunger and therefore is a viable strategy for weight loss in individuals with obesity. Limitations include an increase in protein intake in the study diets (30% of energy from protein) compared to the maintenance diet (15% of energy from protein), which, as noted by the authors, may have an independent effect on energy intake (92). These results contrast with the inpatient feeding study conducted by Hall *et al.* (47). Critical differences that may explain these contrasting results are that Johnstone *et al.* (91) controlled the energy density of both diets and intervened for 2 weeks longer while observing similar increases in ßOHB on the KD. Other methodological differences include an intent for weight loss and only male study participants with obesity. Comparing these two highly controlled feeding trials emphasizes the need for additional research of similar rigor to understand potential biological explanations for the divergent results. Questions arising based on methodological differences include whether differential responses occur during weight loss compared to weight maintenance, between weight status, or with the protein content of the diet.

Several other studies conducted in free-living participants provide insight into the possible effects of ketones on hunger and energy intake during weight loss interventions. Struik *et al.* (93) investigated the effects of a 16-weeks very-low-carbohydrate diet (<50 g carbohydrates/day; 14%, 28%, and 58% of energy from carbohydrates, protein, and fat, respectively) compared to a high-carbohydrate diet (53%, 17%, and 30% of energy from carbohydrates, protein, and fat, respectively) in the context of a 30% energy deficit in study participants with obesity and diabetes. While fasted ratings of appetite were similar between diets, in the fed-state, the high-carbohydrate diet scored higher in fullness and prospective consumption ratings at the fourth week, with fullness remaining higher on the last week. However, since weight changes were similar between diets, it can be concluded that the differences in fullness and prospective consumption ratings were insufficient to substantially affect energy intake. It is noteworthy that despite the prescribed low-energy and low-carbohydrate diet, ßOHB increased by <0.3 mM, suggesting that the energy or carbohydrate intake was beyond the defined goals. Falkenhain *et al.* (94) recently utilized mobile health platforms to assess whether a KD weight loss approach was superior to a non-KD weight loss approach. Weight loss at 12 weeks was greater using the KD approach, thus suggesting that energy intake was lower (and/or energy expenditure higher) on the KD. However, self-reported energy intake was not different between diets, and self-reported total carbohydrate intake in the KD group was ∼100 g CHO per day or ∼20% of total energy intake. Of note, ketone levels assessed by breath acetone were not related to self-reported energy intake but were related to self-reported adherence to the diet program (95). Another long-term study, the DIETFITS trial, compared energy intake between a low-carbohydrate diet and a low-fat diet (11). The intervention did not utilize a macronutrient-based diet prescription but rather implemented behavioral strategies to minimize CHO or fat intake sustainably while emphasizing diet quality. No differences in energy intake were detected at 3, 6, or 12 months, although the extent of ketosis was not reported. Limitations must be considered when interpretating energy intake and appetite data studies conducted in free-living conditions. As with all dietary interventions conducted in free-living conditions, actual dietary intake remains difficult to accurately quantify and likely differs from the diet prescription, while single time-point assessments of plasma ßOHB may not reflect the average ßOHB concentrations over a 24-h time course or throughout the duration of the study. Still, these studies conducted in free-living conditions provide valuable information toward the physiologically relevant effects of KDs on energy intake. Taken together, although the literature remains mixed, most reports suggest ketosis is not related to energy intake during weight loss, and evidence of a causal effect in the conflicting reports is lacking.

In notable contrast, multiple reports by Martins *et al.* (96, 97, 98) have shown that increases in appetite after weight loss occur in concert with reductions in circulating ßOHB even if causality cannot be inferred. The effect of ketosis on energy intake after weight loss may be particularly relevant to long-term weight management, as periods of weight loss are often followed by weight regain. The period after weight loss involves a unique physiological state characterized by multiple factors that affect energy balance, including increased appetite and energy intake. Understanding the effect of KD-induced ketosis in the postweight loss state is critical to the role of ketosis in energy balance. The previously mentioned outpatient study by Ebbeling *et al.* (49) provides the most direct evidence regarding the influence of KDs on appetite during weight loss maintenance. After a 12-weeks weight loss diet, three weight loss maintenance diets were implemented for 4 weeks in a crossover design: a low-fat diet, low glycemic index diet, and a very-low-carbohydrate (i.e., ketogenic) diet. There were no differences in the subjective hunger measured before breakfast between the three diets, despite anticipated differences in ßOHB across diets (99). In summary, in both inpatient settings and free-living conditions, KDs do not reduce appetite or energy intake compared to non-KDs, either during weight stability, weight loss, or weight loss maintenance. Low-calorie KDs (and VLCKDs) remain an effective way to reduce energy intake but no more so than low-calorie non-KD approaches (100).

### Supplementation with MCTs and exogenous ketones

In contrast to KDs and VLCKDs, the intake of MCTs and ketone esters induces ketosis through dietary supplementation that can be applied outside of the context of weight loss or the restriction of dietary carbohydrates. These approaches provide the most relevant data for identifying the independent effects of ketosis on energy intake.

The impact of MCTs on appetite and energy intake has been extensively investigated. A systematic review and meta-analysis by Maher and Clegg (101) indicates that MCTs suppress energy intake compared to LCTs, without affecting subjective measures of appetite or biological indicators of appetite, such as gut peptide concentrations. Seventeen studies with 291 total participants were included in the systematic review, while 11 studies were utilized in the meta-analysis on the effect of MCTs on energy intake. The studies included were conducted in healthy individuals with normal weight, aged 18–70 years, and utilized an MCT intervention with an LCT control arm matched in calories and macronutrient composition, and reported food intake. The meta-analysis revealed a moderate reduction in acute, ad libitum energy intake with MCT consumption (mean effect size [calculated as the standardized difference in means] of −0.444). The average MCT dose used in the studies was 23.8 g in a meal, which based on commonly reported plasma ßOHB concentrations after MCT ingestion can be inferred to have induced an increase in postprandial plasma ßOHB by ∼0.3 mM above baseline. Given that this finding corroborates and builds upon prior systematic reviews and meta-analyses (102, 103, 104) including those conducted on preclinical models (105), the current evidence supports the conclusion that chronic MCT supplementation leads to MCT-induced ketosis, reduces energy intake independent of subjective appetite ratings, and produces modest weight loss. This is further supported by short-term studies that have evaluated the impact of acute MCT ingestion on ad libitum energy intake. A common study design is to provide a standardized breakfast meal containing various amounts of MCTs or LCTs and measure ad libitum food intake at subsequent meals. Van Wymelbeke *et al.* (106) used this approach in combination with four high-carbohydrate diets. Each diet was supplemented with a fat substitute (control) or ∼40 g of either saturated LCTs (lard), unsaturated LCTs (olive oil), or MCTs. The breakfast in the diet supplemented with MCTs increased circulating ßOHB to ∼0.3–0.4 mM (compared to <0.05 mM in the other diets) and resulted in lower energy intake at the ad libitum lunch meal. This reduction in energy intake occurred despite a lack of difference in subjective appetite ratings. When MCTs were supplemented at lunch, energy intake at an ad libitum dinner was reduced, again without any effect on subjective appetite ratings (37). Similarly, reduced energy intake was reported up to 48 h following a breakfast supplemented with 25 g of MCTs that increased circulating ßOHB by ∼0.3 mM (∼25-50% greater than LCT-supplemented breakfasts) (107), again with no differences in subjective appetite or appetite hormones (ghrelin and peptide YY). Notably, these results were not consistently replicated when whole coconut oil was used as the source of MCTs (108) or when implemented outside of the context of a high-carbohydrate meal (109). Overall, the intake of at least 25 g of MCTs reduces energy intake in the subsequent 3–48 h in the context of a high-carbohydrate diet, without affecting subjective appetite. In summary, MCTs induce ketosis in the context of a high-carbohydrate diet, reduce acute energy intake for up to 48 h and lead to modest weight loss with chronic consumption.

Mechanistically, MCTs potentially suppress appetite and hunger by inhibiting the acylation of the orexigenic hormone ghrelin (110, 111), but some reports show that MCTs (and KDs) increase the active form of ghrelin, namely acyl-ghrelin (112, 113). MCT intake may provide MCT moieties that confer various effects on ghrelin activity. Although ghrelin shows a higher affinity for C8-MCTs (114), a difference in activity with MCT-modification of various carbon lengths has not been found. Thus, the effect of MCT moieties of various carbon lengths on ghrelin activity is of critical interest. Another potential mechanism may be through other molecules that impact the ghrelin receptor, as increases in circulating ßOHB were recently shown to directly downregulate liver-expressed antimicrobial peptide-2, an endogenous ghrelin receptor antagonist (115). It also remains possible that gastrointestinal distress or nausea (side effects observed with MCT ingestion) contribute to the reductions in energy intake.

Recently, exogenous ketone esters have gained popularity and provide new insight into the effects of ketones on appetite and energy intake. Stubbs *et al.* (116) utilized a single-blinded crossover study to determine the short-term effects of elevating circulating ketones with a ketone ester drink compared to an energy-matched dextrose (carbohydrate) placebo. Circulating ßOHB reached 3.3 mM 1 hour after the ketone ester drink and remained above 0.5 mM through 4 hours, whereas ßOHB in the placebo condition remained unchanged at ∼0.2 mM. Hunger, desire to eat, and fullness (measured by visual analog scales) indicated reduced appetite with the ketone ester supplement compared to the dextrose control. Consistent biological findings included lower ghrelin as well as correlations between ßOHB concentrations and appetite measures. Other anorexigenic molecules (glucagon-like peptide 1 and peptide YY) also displayed a blunted response with the ketone ester drink. Further evidence from preclinical models show that exogenous ketone esters reduce food intake (117) and cause weight loss (118). However, this has yet to be replicated in humans (119), while adequate study designs to determine the effect of exogenous ketones ester-induced ketosis on acute or chronic energy intake are lacking. Monitored ad libitum feeding trials like those discussed above for MCTs and KDs would provide critical information to address this gap. Additional considerations include the form of exogenous ketones used (along with ketone esters, ketone salts have been used, which have unique pharmacokinetic and pharmacodynamic profiles), ingestion with or without a concomitant meal and adequate blinding to address the poor palatability of exogenous ketone beverages (38). Given the relatively recent utilization of exogenous ketone esters, additional studies in larger, more diverse cohorts are required to corroborate these early findings.

### An integrative view of the effects of ketone bodies on energy intake and appetite

Table 1 summarizes the evidence presented herein about the effects of the various ketosis-inducing strategies on appetite and energy intake in humans. From the available literature, the effects of ketone bodies on energy intake seem contradictory. The reasons for the disagreement between the results from KDs compared to the intake of MCTs and ketone esters are unclear. Differences in the cyclic nature of ketone concentrations and alignment with circadian rhythm and food anticipatory behavior may be involved. Of note, both MCTs and ketone esters are known to induce nausea, as do some of the novel pharmacologic agents for weight management (120). Disentangling the roles of nausea-induced changes in appetite and energy intake versus the role of ketone signaling per se in response to MCTs or ketone esters is difficult. Still, dietary supplement approaches, like MCTs and ketone esters, increase circulating ßOHB and are associated with reduced energy intake and modest weight loss in the context of high carbohydrate availability. In contrast, dietary pattern approaches, like KDs, provide no additional effect on appetite or energy intake compared to non-KD approaches. Together, the overall data suggest ketosis in the context of a carbohydrate-based diet seems to reduce energy intake.

## Impact of Ketosis on Energy Balance and Weight Loss

**Fig. 2:**
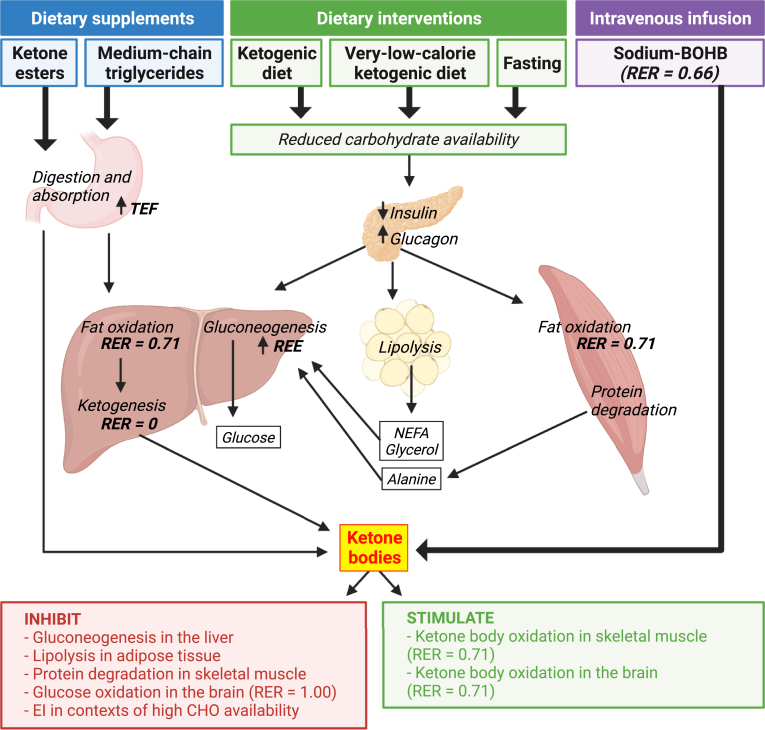
Summary of the effects of ketone bodies on human energy metabolism. Different strategies produce various responses in the organ systems that modify resting energy expenditure (REE) and whole-body substrate utilization (as indicated by the respiratory exchange ratio [RER]) finally leading to ketogenesis. Increased circulating ketones (mostly beta-hydroxybutyrate [BOHB]) then inhibit or stimulate several physiological processes that influence REE, RER, and energy intake (EI). CHO, carbohydrate; NEFA, non-esterified fatty acids; TEF, thermic effect of food.

The evidence presented above discussed how different strategies can induce ketosis and the potential effects on energy expenditure, substrate utilization, and energy intake. Fig. 2 summarizes the physiological responses induced by those strategies and the subsequent effects of circulating ketones. In this section, we integrate this information to gain insight into the effects of ketosis on energy balance and body weight.

The subtle or null influence of KD- and VLCKD-induced ketosis on energy expenditure and energy intake suggests that these strategies would not be uniquely effective for weight loss. In agreement, most long-term weight loss comparisons between KD and non-KD approaches yield equivalent weight loss (121, 122). Less extreme dietary regimens also show that 2-years weight loss is independent of the macronutrient composition in an outpatient context (123). Similarly, Hall and Guo (12) conducted a systematic review and meta-analysis of the effects of isocaloric, isonitrogenous diets with different carbohydrate-to-fat ratios on the rate of fat mass loss in humans. Importantly, only studies in which participants were provided with all foods were included. Therein, the diets with higher fat-to-carbohydrate ratios (i.e, potentially more ketogenic) showed slightly lower rates of fat mass loss (16 g/day of difference). Such a difference was considered physiologically meaningless. These observations suggest that KDs do not influence energy balance differently from non-KDs. In contrast, another systematic review and meta-analysis showed greater long-term weight loss on the implementation of VLCKDs compared to low-fat diets (124). Two critical limitations of this review are worth highlighting, though. First, drastic VLCKDs were compared to low-fat diets which were not necessarily very low in energy. The differences in weight loss may thus result from different energy intake and not from different macronutrient compositions or ketosis. Second, the report did not assess continued ketosis through weight maintenance, thus the potential effect of ketosis on long-term energy balance cannot be established.

As for the intake of MCTs and ketone esters, the enhanced TEF and the acute reductions in energy intake in the context of high-carbohydrate diets suggest these strategies may induce a negative energy balance. A meta-analysis of studies lasting >3 weeks showed that MCT intake was related to modest body weight loss (0.51 kg) and favorable effects on body composition (103). A separate meta-analysis of studies using at least 5 g of MCT ingestion for ≥4 weeks also showed reductions in body weight (0.69 kg) and favorable effects on body composition (104). Finally, the use of MCTs at the onset of a VLCKD accelerated the induction of ketosis and increased weight loss (125). These data may support the role of MCT intake on energy balance and weight loss in free-living individuals even if the impact on weight loss is marginal. The effect of MCTs may be partially explained by the increases in circulating ketones. Note, however, that the role of ketones per se on the components of energy balance has not been supported by careful inpatient studies in which precise measures of food intake, energy expenditure, and energy balance are performed. Using ketone ester supplementation in this type of highly controlled trials may provide valuable information about the influence of ketones on the components of energy balance. The reasons for the differences between free-living and inpatient conditions need to be elucidated but may be simply related to the level of adherence to the prescribed interventions. Careful inpatient studies should determine whether ketones mediate the effects of MCTs on weight loss. If so, ketone bodies would become a valuable target in obesity management.

## Conclusions

In this narrative review, we have discussed the most relevant evidence available to ascertain the independent impact of ketosis on energy expenditure, substrate utilization, energy intake, and the resulting energy balance in humans. Multiple strategies for inducing ketosis were considered: KDs, VLCKDs, fasting, MCT supplementation, exogenous ketone ester supplementation, and intravenously infused ketones. Each strategy provides distinct insight into the role of ketone bodies on energy balance. Importantly, these strategies are accompanied by multiple physiological responses besides increased ketosis. The complexity of the physiological responses makes it difficult to isolate the effects of ketone bodies themselves. This limitation must be considered when interpreting the available evidence. Still, we offer the following conclusions:

• The ketosis induced by KDs and VLCKDs occurs through restricting carbohydrate availability and does not appear to have major effects on energy intake or energy expenditure. Nevertheless, ketosis produces a change in substrate utilization favoring ketone body oxidation.
• Regarding the effect on weight loss, KDs and VLCKDs are equivalent to non-KDs. This is likely due to the null effects of ketosis on whole-body energy balance.
• MCT and ketone ester supplementation independently increase ketones in the context of a high-carbohydrate diet and may decrease energy intake and subsequently lead to a negative energy balance and modest weight loss.
• A combination of highly controlled feeding studies conducted in metabolic wards and fully provisioned feeding trials in free-living conditions are needed to determine the effects of ketone bodies on energy expenditure, substrate oxidation, energy intake, and energy balance. These studies are also required to elucidate the discrepancies between the effects observed with the different approaches for inducing ketosis.

## Data Availability

All the data are contained within the manuscript.

## Conflict of interest

The authors declare that they have no conflicts of interest with the contents of this article.
